# Editorial: Recent advances in artificial neural networks and embedded systems for multi-source image fusion

**DOI:** 10.3389/fnbot.2022.962170

**Published:** 2022-08-03

**Authors:** Xin Jin, Jingyu Hou, Shin-Jye Lee, Dongming Zhou

**Affiliations:** ^1^School of Software, Yunnan University, Kunming, China; ^2^Institute of Technology Management, National Chiao Tung University, Hsinchu, Taiwan; ^3^School of Information Technology, Deakin University, Geelong, VIC, Australia

**Keywords:** artificial neural networks, embedded learning system, feature extraction, fusion strategy, image fusion

Multi-source image fusion can help robotic systems to perceive the real world by fusing multi-source images from multiple sensors into a synthesized image that provides either a comprehensive or reliable description (Geng et al., 2016; Jin et al., 2017; Ma et al., 2017; Liu et al., 2018; Zhu et al., 2018; Zhang et al., 2021). At present, a large number of brain-inspired algorithm methods (or models) are aggressively proposed to accomplish image fusion asksk, and the artificial neural network has become one of the most popular techniques in the field of multi-source image fusion, especially deep convolutional neural networks (Liu et al., 2018; Jin et al., 2021). This is an exciting research field for the research community surrounding image fusion, with deep few-shot learning, unsupervised learning, application of embodied neural systems, and industrial applications.

How to develop a sound biological neural network and embedded system to fuse the multiple features of source images are two key questions that need to be addressed in the field of multi-source image fusion (Liu et al., 2019; Xu and Ma, 2021; Tang et al., 2022). Hence, studies of image fusion can be divided into two areas: first, new end-to-end neural network models for merging constituent parts during the image fusion process; second, the embodiment of artificial neural networks for image fusion systems. In addition, current booming techniques, including deep neural systems and embodied artificial intelligence systems, have been considered potential future trends for reinforcing the performance of image fusion.

In the first work entitled “Multi-Focus Color Image Fusion Based on Quaternion Multi-Scale Singular Value Decomposition (QMSVD)”, Wan et al. employed multichannel quaternion multi-scale singular value to decompose the multi-focus color images, and a set of low-frequency and high-frequency sub-images was obtained. The activity and matching levels are exploited in the focus decision mapping of the low-frequency sub-image fusion, and a local contrast fusion rule based on the integration of high-frequency and low-frequency regions was also proposed. The fused images were finally reconstructed by inverse QMSVD. Experiments revealed that the color image fusion method has competitive visual effects.

The visual quality of images is seriously affected by bad weather conditions, especially on foggy days. To remove the fog in the image, Liu et al. introduced a method entitled “Single Image Defogging Method Based on Image Patch Decomposition and Multi-Exposure Image Fusion”. In this method, the authors propose a single image defogging method based on image patch decomposition and multi-exposure fusion, which did not use any a priori knowledge of the scene depth information. First, a single foggy image was processed to produce a set of underexposed images, and then the underexposed and original images were enhanced and fused by guided filter and patch operation.

To protect the Tujia brocades that form part of the intangible cultural heritage, Shuqi He introduce a method using an unsupervised clustering algorithm for Tujia brocades segmentation, and a K auto-selection based on information fusion was also used. In this method, the cluster number K was calculated by fusing local binary patterns and gray-level co-occurrence matrix characteristic values. Thus, the clustering and segmentation operation can be performed on Tujia brocade images by adopting a Gaussian mixture model to get a rough preliminary segmentation image. Then, the voting optimization and conditional random filtering operation were used to optimize the preliminary segmentation and produce the final result.

In the fourth paper, Wu et al. propose fractional wavelet-based generative scattering networks (FrScatNets) in which fractional wavelet scattering networks are used as the encoder to extract image features, with deconvolutional neural networks acting as the decoder, to generate an image. Moreover, the authors also developed a feature-map fusion method to reduce the dimensionality of FrScatNet embeddings. In this work, the authors also discuss the application of image fusion in this study.

Conventional tensor decomposition is a kind of approximate decomposition model in which the image details may be lost in fused image reconstruction. To overcome this problem, Lu et al. introduced a work entitled “multi-modal image fusion based on matrix product state of tensor”. In this work, source images were first separated into a third-order tensor, so that the tensor can be decomposed into a matrix product form by singular value decomposition, and then the Sigmoid function can be employed to fuse the key components. Thus, the fused image can be reconstructed by multiplying all the fused tensor components.

Lin et al. introduced an integrated circuit board object detection and image augmentation fusion model based on YOLO. In this paper, the authors first analyzed several popular region-based convolutional neural networks and YOLO models, and then they proposed a real-time image recognition model for integrated circuit board (ICB) in the manufacturing process. In this work, the authors first constructed an ICBs training dataset, and a preliminary image recognition model was then established to classify and predict ICBs. Finally, image augmentation fusion and optimization methods were used to improve the accuracy of the method.

Yu et al. report on a bottom-up visual saliency model in the wavelet domain. In this method, wavelet transform was first performed on the image to achieve four channels, and then discrete cosine transform was used to get the magnitude spectra and corresponding signum spectra. Third, wavelet decomposed multiscale magnitude spectra for every single channel were produced. Fourth, six multiscale conspicuity maps were generated for every single channel, and then the multiscale conspicuity maps of the four channels were fused. At last, a final saliency map after a scale-wise combination was obtained. The experimental results showed that the proposed model is effective.

Shi et al. propose an ensemble model for graph networks on imbalanced node classification, which uses GNNs as the base classifiers during boosting. In this method, the higher weights were set for the training samples that were not correctly classified by the previous classifiers. Besides, transfer learning was also employed to reduce computational cost and increase fitting ability. Experiments showed that the proposed method can achieve better performance than a graph convolutional network.

Deep neural networks have proven vulnerable to attack from adversarial examples. In response, Xie et al. propose a new noise data enhancement method, which only transforms adversarial perturbation to improve the transferability of adversarial examples with noise data enhancement and random erasing. Experiments have proved the effectiveness of this method.

The GAN-based method is difficult to converge completely to the distribution of face space in training. Yang et al. propose a face-swapping method based on a pretrained StyleGAN generator and designed a control strategy of the generator based on the idea of encoding and decoding to overcome the problem of GAN in this task. Experiments have shown that the performance of the proposed method is better than other state-of-the-art methods.

In the paper entitled “Adaptive fusion based method for imbalanced data classification”, Liang et al. propose an ensemble method that combines data transformation and an adaptive weighted voting scheme for imbalanced data classification. They first utilized modified metric learning to obtain a feature space based on imbalanced data, and then the base classifiers were assigned different weights, adaptively. Experiments on multiple imbalanced datasets were performed to verify the performance of this algorithm.

In the work entitled “Multi-Exposure Image Fusion Algorithm Based on Improved Weight Function”, Xu et al. proposed a multi-exposure image fusion method based on the Laplacian pyramid. Based on the Laplacian pyramid decomposition, an improved weight function was used to capture source image details. Six multi-exposure image fusion methods were compared with the proposed method on 20 sets of multi-exposure image sequences.

Sketch face recognition can match cross-modality facial images from sketch to photo, which is important in criminal investigations. Guo et al. introduced an effective cross task modality alignment network for sketch face recognition, and a meta learning training episode strategy was introduced to address the small sample problem. In this work, they propose a two-stream network to capture modality-specific and sharable features, and two cross task memory mechanisms to improve the performance of feature learning. At last, a cross task modality alignment loss is proposed to train the model.

## Author contributions

All authors listed have made a substantial, direct, and intellectual contribution to the work and approved it for publication.

## Funding

This work was supported in part by the National Natural Science Foundation of China (Nos. 62101481 and 62002313), the Basic Research Project of Yunnan Province (Nos. 202201AU070033 and 202201AT070112), and the Key Laboratory in Software Engineering of Yunnan Province (No. 2020SE408).

## Conflict of interest

The authors declare that the research was conducted in the absence of any commercial or financial relationships that could be construed as a potential conflict of interest.

## Publisher's note

All claims expressed in this article are solely those of the authors and do not necessarily represent those of their affiliated organizations, or those of the publisher, the editors and the reviewers. Any product that may be evaluated in this article, or claim that may be made by its manufacturer, is not guaranteed or endorsed by the publisher.

## References

[B1] Geng P. Huang M. Liu S. Feng J. Bao P. (2016). Multifocus image fusion method of Ripplet transform based on cycle spinning. Multimedia Tools Applic. 75, 10583–10593. 10.1007/s11042-014-1942-1

[B2] Jin X. Huang S. Jiang Q. Lee S. J. Wu L. Yao S. (2021). Semi-Supervised Remote Sensing Image Fusion Using Multi-Scale Conditional Generative Adversarial network with Siamese Structure. IEEE J. Select Topics Appl. Earth Observ. Remote Sensing 14, 7066–7084. 10.1016/j.inffus.2017.10.007

[B3] Jin X. Jiang Q. Yao S. Zhou D. Nie R. Hai J.. (2017). A Survey of Infrared and Visual Image Fusion Methods. Infrar. Phys. Technol. 85, 478–501. 10.1016/j.infrared.2017.07.010

[B4] Liu S. Wang J. Lu Y. Hu S. Ma X. Wu Y. (2019). Multi-focus image fusion based on residual network in non-subsampled shearlet domain. IEEE Access. 7, 152043–152063. 10.1109/ACCESS.2019.2947378

[B5] Liu Y. Chen X. Wang Z. Wang Z. J. Ward R. K. Wang X. (2018). Deep learning for pixel-level image fusion: Recent advances and future prospects. Inf. Fusion 42:158–173. 10.1109/JSTARS.2021.3090958

[B6] Ma K. Li H. Yong H. Wang Z. Meng D. Zhang L. (2017). Robust multi-exposure image fusion: a structural patch decomposition approach. IEEE Trans. Image Proc. 26, 2519–2532. 10.1109/TIP.2017.2671921 28237928

[B7] Tang L. Yuan J. Ma J. (2022). Image fusion in the loop of high-level vision tasks: A semantic-aware real-time infrared and visible image fusion network. Inf. Fusion 82, 28–42. 10.1016/j.inffus.2021.12.004

[B8] Xu H. Ma J. (2021). EMFusion: An unsupervised enhanced medical image fusion network. Inf. Fusion 76, 177–186. 10.1016/j.inffus.2021.06.001

[B9] Zhang H. Xu H. Tian X. (2021). Image fusion meets deep learning: A survey and perspective. Inf. Fusion 76, 323–336. 10.1016/j.inffus.2021.06.008

[B10] Zhu P. Ding L. Ma X. Huang Z. (2018). Fusion of infrared polarization and intensity images based on improved toggle operator. Optics Laser Technol. 98, 139–151. 10.1016/j.optlastec.2017.07.054

